# Healthcare Resource Utilization and Economic Outcomes of RSV-Hospitalized Patients Aged ≥ 60 Years: A Retrospective Cohort Study

**DOI:** 10.3390/diseases13030068

**Published:** 2025-02-21

**Authors:** Anna Puggina, Melania Dovizio, Alexander Domnich, Alen Marijam, Chiara Veronesi, Caterina Rizzo, Marta Vicentini, Luca Degli Esposti, Giovanna Elisa Calabrò, Maria João Fonseca

**Affiliations:** 1 GSK, 37135 Verona, Italy; 2 CliCon S.r.l. Società Benefit, Health, Economics & Outcomes Research, 40137 Bologna, Italy; 3 Hygiene Unit, San Martino Policlinico Hospital-IRCCS for Oncology and Neurosciences, 16132 Genoa, Italy; 4 GSK, 1300 Wavre, Belgium; 5 Department of Translational Research and New Technologies in Medicine, Surgery University of Pisa, 56126 Pisa, Italy; 6 VIHTALI (Value in Health Technology and Academy for Leadership & Innovation), Spin-Off of Università Cattolica del Sacro Cuore, 00168 Rome, Italy; 7 Department of Human Sciences, Society and Health, University of Cassino and Southern Lazio, 03043 Cassino, Italy; 8 GSK, 1495-136 Lisbon, Portugal

**Keywords:** respiratory syncytial virus (RSV), older adults, high risk, hospitalization, economic burden, healthcare costs

## Abstract

Background/Objectives The economic impact of respiratory syncytial virus (RSV) in Italy is not well defined. This analysis assessed the economic outcomes of RSV-hospitalized patients aged ≥ 60 years in Italy. Methods: Healthcare resource utilization and direct healthcare costs during the first RSV hospitalization and 12-month follow-up were collected from Italian administrative databases. A propensity-score-matched (PSM) analysis was performed between patients hospitalized for RSV and those hospitalized for any cause (without an RSV diagnosis). Results: Among 201 patients, an average of 1.95 hospitalizations, 19.38 prescriptions, and 7.11 outpatient services were reported during the first RSV hospitalization and the following 12 months. The mean direct healthcare costs were EUR 11,599 (related to hospitalization [79%], prescriptions [16%], and outpatient services [5%]). Following PSM analyses, direct healthcare costs were 15% higher for RSV-hospitalized patients versus those hospitalized for any cause (EUR 9369 versus EUR 8173; *p* < 0.05), driven by differences in hospitalizations (EUR 7477 versus EUR 6327; *p* < 0.05) and intensive care unit admissions (EUR 818 versus EUR 178; *p* = 0.001). Conclusions: Despite a limited sample size, this study reports a substantial economic burden associated with RSV-hospitalized patients aged ≥ 60 years in Italy. The results provide important evidence to inform preventative RSV strategies to reduce the economic burden on the Italian National Health Service.

## 1. Introduction

Respiratory syncytial virus (RSV) is a major cause of acute respiratory infection. While RSV infections are common in adults, most individuals experience mild symptoms and therefore cases may not be detected or officially diagnosed [1]. However, in some cases, infection with RSV can lead to severe disease and complications, such as exacerbation of pre-existing comorbidities, hospitalization, and death [2,3]. Moreover, the risk of developing severe complications following RSV infection increases with age, immunodeficiency conditions, and comorbidities, including lung disease, heart disease, and diabetes [4,5].

Alongside an increased risk of more serious complications, RSV infection in older adults or those with comorbidities is associated with a high clinical burden, including a higher number of hospital stays and intensive care unit (ICU) admissions [3,6]. The annual burden of acute respiratory infection (ARI) caused by RSV (RSV-ARI) in high-income countries (United States, Canada, European countries, Japan, and South Korea) among those aged ≥ 60 years has been estimated at over 5,000,000 RSV-ARI cases, nearly 500,000 hospitalizations, and over 30,000 in-hospital deaths [7]. In addition, RSV has been estimated to cause 4.66% and 7.03% of annual symptomatic respiratory infections among older and high-risk adults, respectively, in high-income countries [2]. Despite a seemingly high incidence, RSV is potentially underreported; a systematic literature review of the burden of ARI in older adults in high-income countries previously estimated a pooled annual RSV-ARI hospitalization rate in those aged ≥ 65 years of 157 per 100,000 population [8]. However, after this value was adjusted for under-ascertainment of RSV, the annual incidence was approximately 2.2 times higher [8]. The assumed underestimation of RSV burden among older adults also occurs at a national level in Italy, where the existing RSV surveillance framework relies on influenza-like illness (ILI)-based and fever-based case definitions [9,10]. This approach overlooks many mild-to-moderate RSV cases in adults that do not present with fever, potentially resulting in an underestimation of RSV-ARI cases by up to nine-fold [11]. Additional challenges leading to under-reporting and under-ascertainment of RSV include the suboptimal sensitivity of several diagnostic tests, lack of uniformity in RSV testing, and misclassification of International Classification of Diseases (ICD) codes [8,12,13]. Precise case definitions and enhanced diagnostic strategies are essential to accurately estimate the epidemiological impact of RSV in Italy. Pooled estimate data showed that 4.5% of respiratory samples tested positive for RSV in Italian adults, with higher rates in older adults (4.4%) than in working age adults (3.5%) [9]. With an aging population in Italy, the prevalence of chronic diseases such as respiratory diseases, diabetes, and cardiac pathology is increasing [14]. With the increased risk of RSV hospitalization in older-aged adults and those with comorbidities, the aging population and increased prevalence of chronic diseases in Italy highlights the need for appropriate and effective preventative RSV strategies [15,16].

The clinical burden described above is also associated with an economic burden, including high levels of healthcare resource utilization (HCRU) and high direct healthcare costs [17,18,19]. However, a recent Italian systematic literature review on the epidemiology and burden of RSV among older adults was unable to find studies reporting RSV complication and hospitalization rates in Italy [9]. This suggests that the overall economic burden associated with RSV on the Italian National Health Service (NHS) is unknown.

The primary objective of this study was to estimate all-cause HCRU and direct healthcare costs, covered by the Italian NHS, for RSV-hospitalized patients aged ≥ 60 years through a 12 month follow-up period, stratified by age group, comorbidities, and immunocompromised status. The secondary objective was to compare all-cause HCRU and direct healthcare costs in RSV-hospitalized patients with a matched population of patients hospitalized for any cause.

## 2. Materials and Methods

### 2.1. Data Source

This observational retrospective analysis used data from six Italian administrative databases, providing pooled information from Italian local health authorities (LHAs), geographically distributed across 11 out of 20 Italian regions and covering approximately 20% of the entire Italian population. The population retrieved by the databases was deemed representative, in terms of age and sex, of the whole Italian population [20,21]. The data were initially collected for and used by the Italian NHS for the reimbursement of healthcare services, but these databases have been previously validated for conducting cost analyses.

The administrative databases used in this analysis included a beneficiary database, containing demographic information on all included patients; a pharmaceutical database, containing prescription information on all included patients; a hospitalization database, containing hospitalization data for all included patients; a diagnostic test and specialist/outpatient visit database, containing information on tests and visits for all included patients; a patient waiver database, containing active payment waiver codes; and a laboratory test result database, containing laboratory results for a subsample of LHAs.

To ensure that patient privacy was maintained in full compliance with the European General Data Protection Regulation (GDPR; 2016/679), an anonymous univocal numeric code was assigned to each included participant. The patient’s code in each database allowed for electronic linkage between all databases. No patient identifiers were provided to the authors. All results of the analyses were presented as aggregated summaries, preventing them from being directly or indirectly attributed to individual patients.

### 2.2. Study Design and Patient Population

Patients aged ≥ 60 years at the index date, with at least one record of hospitalization with a primary or secondary discharge diagnosis of RSV (comprising hospitalization due to RSV [ICD-9-CM code 079.6], acute RSV bronchiolitis [ICD-9-CM code 466.11], or RSV pneumonia [ICD-9-CM code 480.1]) from 1 January 2010 to 31 December 2021, were included. The index date was defined as the date of the first RSV hospitalization discharge diagnosis code and patients were followed up for a 12-month period from the index date (Supplementary Figure S1). To be included in this study, the patients needed to satisfy the following additional criteria: (i) at least 12 months of data availability before and after the index date, and (ii) complete linkage between all databases. For comparison, a control cohort was included, and defined as individuals with at least one hospitalization (any cause) and without a record of RSV hospitalization or a positive RSV laboratory test result during the study period. Patients who transferred to a different LHA during the study period were excluded from the analysis to prevent missing data.

### 2.3. Data Collection and Analysis

Patient demographics (age and sex) at the index date and clinical characteristics (Charlson Comorbidity Index [CCI], risk status, presence of comorbidities, and immunocompromised status) during the baseline period (defined as the all-available period before the index date) were extracted from the six administrative databases. CCI is a validated index used to predict one-year mortality, and comorbidity data for the year prior to the index date were reported as low (0), medium (1–2), high (3–4), and very high (≥5) CCI scores. All-cause HCRU and direct costs covered by the Italian NHS during the first RSV hospitalization (index hospitalization) and the 12 months following RSV hospitalization were also collected. All-cause HCRU and direct healthcare costs included hospitalizations (all-cause hospitalizations, RSV-related admissions, and ICU admissions), prescriptions for all medications, and specialized outpatient services (including specialist visits, diagnostic, and laboratory tests).

The patients were stratified by age (≥60 years, ≥65 years, and ≥75 years), whether they were high-risk, had comorbidities (chronic obstructive pulmonary disease [COPD], heart disease, and diabetes), and whether they were immunocompromised. The patients were defined as high-risk if they had ≥1 comorbidity of interest during the baseline period. Comorbidities of interest included COPD, asthma, other chronic respiratory/pulmonary disease, diabetes, heart failure, advanced liver disease, renal disease, and chronic kidney disease. The patients were defined as immunocompromised by the presence of at least one of the following conditions at baseline: hematopoietic stem cell transplant (HSCT), solid organ transplantation, chronic inflammatory diseases, human immunodeficiency virus (HIV), end-stage renal disease (ESRD), cancer, and immunosuppressive therapy. The codes and criteria for the identification of comorbidities and immunocompromised patients are shown in Supplementary Table S1.

Direct healthcare costs were reported in Euros (EUR). Hospitalization costs were determined using Diagnosis-Related Group tariffs, representing Italian NHS reimbursement levels to healthcare providers. Drug prescription costs were evaluated using the NHS purchase price. The costs of instrumental and laboratory tests were defined according to tariffs applied by each region.

To analyze the impact of RSV infections and hospital admissions on the Italian NHS, a comparative analysis was conducted to compare all-cause HCRU and direct healthcare costs in RSV-hospitalized patients with a matched population of patients hospitalized for any cause (Supplementary Figure S1). The analysis considered patients aged ≥ 60 years hospitalized for RSV and patients aged ≥ 60 years hospitalized for any cause (control cohort). A propensity score was estimated using logistic regression, considering age, sex, geographic area of residence, and year of index hospitalization. The patients were then stratified into quintiles based on their propensity score and patients with similar propensity scores were matched at a 1:5 ratio. To examine the balance of covariate distribution in the two cohorts after propensity score matching (PSM), *p*-values and standardized mean difference were evaluated. After PSM, there were no significant differences between RSV-hospitalized individuals and the control cohort in terms of age, sex, risk status, or whether individuals were immunocompromised.

Continuous data were reported as mean and standard deviation (SD). HCRU data are reported with outliers included and direct healthcare costs are reported with outliers excluded. Outliers were defined as values falling ≥3 SDs from the mean. Student’s *t*-test was used to compare the means between two groups at a 5% significance level (*p* < 0.05). Data were analyzed using STATA SE version 17.0 (StataCorp LLC, College Station, TX, USA).

The results of this analysis were presented in tabular form and aggregate analyses omitted patient identification, meaning that informed consent was not required.

## 3. Results

### 3.1. Demographic and Clinical Characteristics

In total, 201 patients aged ≥ 60 years with at least one hospitalization for RSV between 1 January 2010 and 31 December 2021 were identified and included. At the index date, the mean (SD) age was 77.8 (10.4) years and 61.2% of patients were female. A high and very high CCI was observed in 20.4% and 6.5% of patients, respectively, and the most common pre-existing conditions were COPD (40.3%), diabetes (23.9%), and heart failure (17.4%). Furthermore, 42.3% of patients were immunocompromised. Demographics and clinical characteristics stratified by age are presented in Supplementary Table S2; the same characteristics stratified by pre-existing comorbidities and immunocompromised status are presented in Supplementary Table S3.

### 3.2. Economic Outcomes for RSV-Hospitalized Patients

#### 3.2.1. Overall HCRU and Direct Healthcare Costs Among RSV-Hospitalized Patients

**Figure 1:**
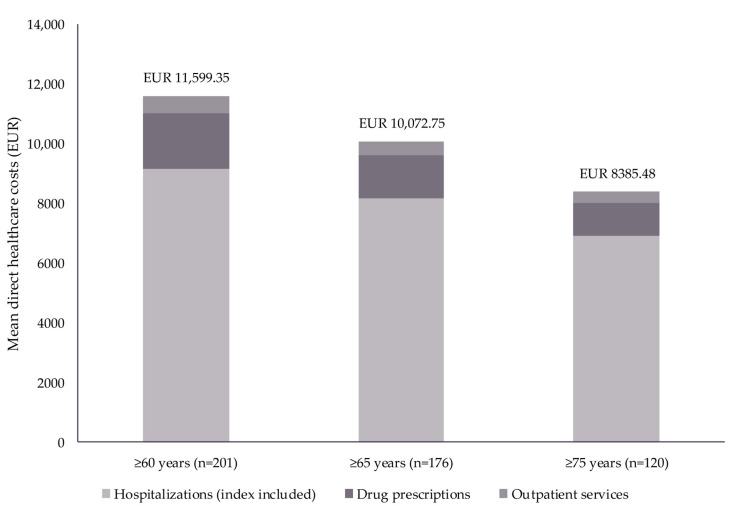
Mean direct healthcare costs per patient during the index hospitalization and 12-month follow-up period, stratified by age. Data reported as mean in Euros (EUR) with outliers (defined as values that fall ≥3 SDs from the mean) excluded. A full breakdown of direct healthcare costs is reported in Table 2. Abbreviations: SD, standard deviation.

Mean HCRU during the index hospitalization and 12-month follow-up period, stratified by age, is summarized in Table 1; mean direct healthcare costs stratified by age are reported in Table 2 and Figure 1.

The total mean direct healthcare costs during the 12-month follow-up were EUR 11,599 (per patient aged ≥ 60 years hospitalized at least once for RSV) and were mostly related to hospitalizations (79%), followed by prescriptions (16%) and specialized outpatient services (5%). Direct healthcare costs were EUR 10,073 among those aged ≥ 65 years and EUR 8385 among those aged ≥ 75 years (Table 2; Figure 1).

**Figure 2:**
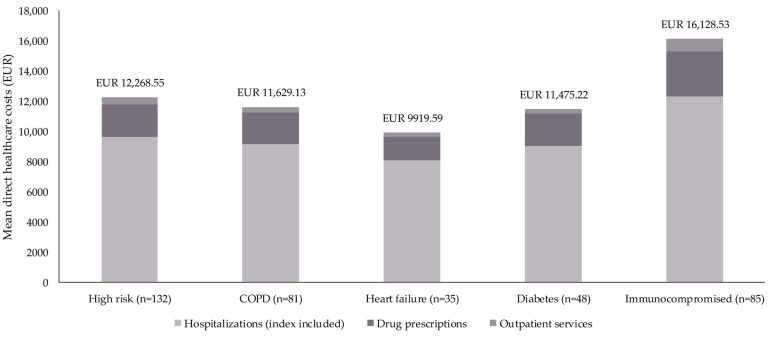
Mean direct healthcare costs per patient during the index hospitalization and 12-month follow-up period, stratified by risk status, comorbidity, and immunocompromised status. Data reported as mean in Euros (EUR) with outliers (defined as values that fall ≥3 SDs from the mean) excluded. A full breakdown of direct healthcare costs is reported in Table 4. Abbreviations: COPD, chronic pulmonary obstructive disorder; SD, standard deviation.

Mean HCRU, stratified by whether patients were high-risk, had comorbidities, or were immunocompromised, is presented in Table 3; mean direct healthcare costs for these patients are reported in Table 4 and Figure 2.

Total mean direct healthcare costs were EUR 12,269 per high-risk patient. Among patients with specific comorbidities, total mean direct healthcare costs were EUR 11,629 (COPD), EUR 9920 (heart failure), and EUR 11,475 (diabetes) per patient. Mean direct healthcare costs per immunocompromised patient were EUR 16,129 (Table 4; Figure 2).

#### 3.2.2. Hospitalizations Among RSV-Hospitalized Patients

Including the index hospitalization, a mean (SD) of 1.95 (1.6) hospitalizations were reported per patient aged ≥ 60 years during the 12-month follow-up, amounting to EUR 9157 per patient. Patients aged ≥ 65 years and ≥75 years had a mean (SD) of 1.85 (1.4) and 1.73 (1.2) hospitalizations, costing EUR 8163 and EUR 6918, respectively (Table 1 and Table 2; Figure 1). Regarding specific types of hospitalization, patients aged ≥ 60 years, ≥65 years, and ≥75 years had a mean (SD) of 0.05 (0.2) ICU admissions, with these admissions leading to direct healthcare costs of EUR 793 (≥60 years), EUR 625 (≥65 years), and EUR 794 (≥75 years) per patient (Table 1 and Table 2).

Patients aged ≥ 60 years who were considered high-risk had a mean (SD) of 1.99 (1.7) hospitalizations, costing EUR 9615 per patient (Table 3 and Table 4; Figure 2). When patients aged ≥ 60 years were stratified by a specific comorbidity, the mean (SD) number of hospitalizations was 1.91 (1.6) for patients with COPD (costing EUR 9176), 2.09 (1.9) for patients with heart failure (costing EUR 8097), and 1.90 (1.4) for patients with diabetes (costing EUR 9045; Table 3 and Table 4).

Immunocompromised patients aged ≥ 60 years had a mean (SD) of 2.41 (2.1) hospitalizations, with associated direct healthcare costs of EUR 12,356 per patient (Table 3 and Table 4; Figure 2). Specifically, ICU admissions led to a mean direct healthcare cost of EUR 821 per immunocompromised patient, while RSV hospitalizations cost EUR 5701 (Table 3 and Table 4).

#### 3.2.3. Prescriptions Among RSV-Hospitalized Patients

A mean (SD) of 19.38 (17.5) drug prescriptions was reported for patients aged ≥ 60 years, costing EUR 1879 per patient. Mean (SD) prescriptions were 17.00 (17.2) for those aged ≥ 75 years, costing EUR 1081 per patient (Table 1 and Table 2; Figure 1).

High-risk patients aged ≥ 60 years had a mean (SD) of 20.77 (19.2) drug prescriptions, costing EUR 2187 per patient. Stratified by comorbidity, the mean (SD) number of drug prescriptions was 21.28 (19.6) for patients with COPD, 24.63 (21.5) for those with heart failure, and 21.88 (19.7) for patients with diabetes. The direct healthcare costs associated with drug prescriptions for these patients were EUR 2076 (COPD), EUR 1538 (heart failure), and EUR 2149 (diabetes; Table 3 and Table 4; Figure 2).

Similar to hospitalizations, mean direct healthcare costs associated with drug prescriptions were highest among immunocompromised patients, costing EUR 2957 per patient. A mean (SD) of 21.21 (19.0) prescriptions was reported for immunocompromised patients (Table 3 and Table 4; Figure 2).

#### 3.2.4. Outpatient Services Among RSV-Hospitalized Patients

For patients aged ≥ 60 years, the mean (SD) number of outpatient services was 7.11 (11.7) during the 12-month follow-up period. Outpatient services included specialized outpatient visits (mean [SD] for patients aged ≥ 60 years: 2.09 [4.7]) and diagnostic/laboratory tests (mean [SD] for patients aged ≥ 60 years: 5.02 [7.8]). The direct healthcare costs associated with these services amounted to EUR 563 (specialized visits: EUR 47; diagnostic/laboratory tests: EUR 516) per patient (Table 1 and Table 2; Figure 1).

The mean (SD) number of outpatient services was 6.91 (11.9) for high-risk patients, costing EUR 467 per patient. When stratified by comorbidity, the mean (SD) number of outpatient services was 5.26 (6.8) for patients with COPD (costing EUR 377), 5.57 (7.8) for patients with heart failure (costing EUR 284), and 5.42 (6.4) for patients with diabetes (costing EUR 282). Immunocompromised patients had an average of 9.75 (15.5) outpatient services during the index hospitalization and 12-month follow-up, costing EUR 816 per patient (Table 3 and Table 4; Figure 2).

### 3.3. Economic Outcomes for RSV-Hospitalized Patients Versus Patients Hospitalized for Any Cause (Propensity-Score-Matched Analysis)

**Figure 3:**
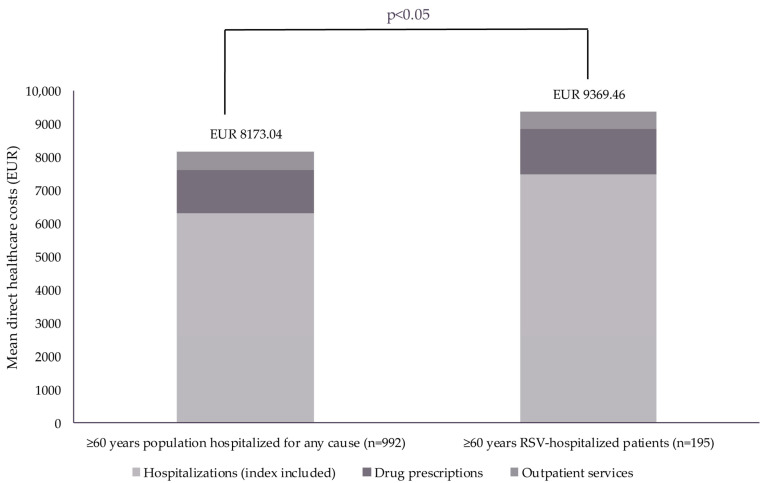
Mean direct healthcare costs among patients hospitalized for RSV and patients hospitalized for any cause (propensity-score-matched analysis). Data reported as mean in Euros (EUR) with outliers (defined as values that fall ≥ 3 SDs from the mean) excluded. A full breakdown of direct healthcare costs is reported in Table 5. Abbreviations: RSV, respiratory syncytial virus; SD, standard deviation.

The PSM analysis showed that the total mean direct healthcare costs were significantly higher for patients hospitalized for RSV (EUR 9369) compared with those hospitalized for any cause (EUR 8173; *p* < 0.05; Table 5; Figure 3).

#### 3.3.1. Hospitalizations (Propensity-Score-Matched Analysis)

The mean number of hospitalizations during the 12-month follow-up period, index hospitalization included, was significantly higher in those hospitalized for RSV versus the control cohort (1.95 versus 1.67; *p* < 0.01); a significant difference was also observed between the direct healthcare costs associated with hospitalizations (EUR 7477 versus EUR 6327; *p* < 0.05). Similarly, mean ICU admissions were higher and associated with higher mean direct healthcare costs among patients hospitalized for RSV versus the control cohort (HCRU: 0.05 versus 0.02 [*p* < 0.05]; direct healthcare costs: EUR 818 versus EUR 178 [*p* = 0.001]; Table 5).

#### 3.3.2. Drug Prescriptions (Propensity-Score-Matched Analysis)

There were no significant differences in the number of drug prescriptions or the direct healthcare costs associated with these prescriptions between patients hospitalized for RSV versus the control cohort (Table 5).

#### 3.3.3. Outpatient Services (Propensity-Score-Matched Analysis)

There were no significant differences in the number of outpatient services or direct healthcare costs associated with these services between patients hospitalized for RSV versus the control cohort (Table 5).

## 4. Discussion

RSV infection in older adults or those with comorbidities can result in severe complications leading to hospitalization and even death [4,22,23]. The clinical complications of the disease are also associated with an economic burden that can place substantial pressure on healthcare systems [24]. This study reports high HCRU and direct healthcare costs among individuals aged ≥ 60 years in Italy during the 12 months after RSV hospitalization (index hospitalization included). Using a PSM approach, this analysis also reported that direct healthcare costs were 15% higher in those hospitalized for RSV versus those hospitalized for any cause and without a diagnosis for RSV.

In this analysis, the mean direct healthcare costs during the 12-month follow-up period amounted to EUR 11,599 per patient and appeared to decrease slightly with increasing age. While seemingly unexpected given that RSV symptoms tend to be more severe with older age, this is a finding also observed elsewhere, including in one study comparing individuals aged 18–49 years with those aged 50–64 years and ≥65 years, which reported a similar observation [25]. Choi et al. attributed the lower costs observed with increasing age partly to length of stay being lower in older patients [25]. Additionally, older adults with RSV may have a higher mortality rate than younger adults, leading to lower direct healthcare costs. As length of stay and mortality rate were not captured in the present analysis, further research is required to understand this observation, the severity of RSV symptoms, and admittance to hospital. Given that only mean direct healthcare costs were calculated in our analysis and SDs were large, further analyses are required before definitive trends can be determined.

It is well established that older adults constitute a large proportion of RSV-hospitalized individuals [16] and these hospitalizations are associated with a substantial economic burden, evidenced by hospitalizations accounting for 79% of direct healthcare costs in this analysis. Understanding the severity of this burden, during both the index hospitalization and subsequent hospitalizations, could help decision-makers define adequate health policies for the management of patients, prevention of disease, and sustainability of the healthcare system. However, European data reporting the direct healthcare costs associated with hospitalizations among older adults hospitalized for RSV are limited. In a study conducted in Germany between 2010 and 2019 (a similar study period to the present analysis), the mean direct hospitalization costs for RSV-hospitalized individuals aged >59 years were EUR 5731 [26]. Despite these costs only accounting for an index hospitalization and not a follow-up period as in the present analysis, this finding corroborates the substantial economic burden associated with hospitalization for RSV [26]. Over a longer time period, among patients hospitalized for influenza in Italy, hospitalizations in the six months after the index hospitalization accounted for 95% of the total healthcare costs [27]. Additionally, patients aged ≥ 65 years and patients with ≥1 comorbidity accounted for over 47% and 52% of hospitalization costs, respectively, furthering the notion that older adults and those considered high-risk are associated with higher costs [27].

In this study, direct healthcare costs were mostly associated with hospitalization across all ages and comorbidities (in both the RSV and propensity-score-matched control cohorts). In comparison with a control cohort, individuals hospitalized for RSV had significantly more hospitalizations and higher direct healthcare costs during the index hospitalization and 12-month follow-up period. Two studies, both conducted in North America, have also reported higher healthcare costs for older adults hospitalized for RSV versus control groups [28,29]. Finally, in the present study, a mean of nearly 20 prescriptions during both the index hospitalization and the following 12 months was reported per patient, costing nearly EUR 1900. In fact, prescriptions alone accounted for 16% of direct healthcare costs. High costs associated with prescriptions among RSV-hospitalized patients are also reported elsewhere [28]. Although the type of drug prescribed was not the focus of this analysis, antibiotics are often prescribed to patients with RSV, even without a secondary bacterial respiratory tract infection [30]. Reducing the need for prescription of antibiotics through RSV preventative strategies, such as vaccination, may help to reduce not only the economic burden associated with the disease but also the development of antimicrobial resistance [31].

This study is associated with some limitations. Firstly, few RSV cases were found, with the database only capturing 201 patients aged ≥ 60 years who were hospitalized at least once for RSV. As there were previously no vaccines for RSV and there are no specific treatments for older adults, there has been a limited need to identify RSV. Therefore, it is likely that no specific ICD codes were used at diagnosis. This lack of codes may result in the underreporting of RSV; indeed, this underreporting has been widely established in the literature [8,10,13]. The requirement for patients to have at least 12 months of data before and after the index date, along with the need for complete database linkage, may have also contributed to this small sample size. However, since the purpose of this analysis was to describe the economic burden per patient with RSV, and given that these databases have been previously validated for cost analyses, it is likely that these results are reflective of the economic burden. Secondly, these data were originally collected for the purpose of reimbursement of healthcare services, rather than for conducting outcomes research, and are subject to coding errors. However, the databases used have been previously validated for the purpose of cost analyses. A further limitation is related to the comparative analysis between PSM cohorts. These cohorts were balanced using a PSM-based approach. While PSM is a valuable methodology to mimic the effect of randomization with retrospective data, it is based on the covariates extracted from the database and evaluated at baseline. Some factors not captured in the databases were not included and the impact of these residual confounders was not considered.

Overall, this study highlights the need to promote public awareness regarding RSV transmission and preventative strategies in Italy to reduce the substantial economic burden currently placed on the NHS. Preventative strategies, such as vaccination against RSV, offers considerable benefits in adults aged ≥ 60 years, with three vaccines approved in Europe to protect older adults against RSV-associated lower respiratory tract disease [32,33,34]. Health authorities and scientific societies across several countries have approved and recommended RSV vaccinations, including the United States, United Kingdom, France, and Germany [35,36,37,38]. The Vaccination Calendar for Life, an alliance of scientific and professional societies of public health physicians, pediatricians, and general practitioners in Italy, recommends RSV vaccination for those aged ≥ 75 years and those aged ≥ 60 years who are at high risk for RSV [39]. Implementing effective vaccination programs for older adults and those with comorbidities could be key to increasing vaccine uptake and thereby decreasing morbidity, mortality, and healthcare costs associated with RSV. Improved surveillance is also required and there is a need to develop and reinforce RSV surveillance systems in Italy that look for ARI, rather than the current ILI-based system [10]. Combining improved knowledge, prevention, and surveillance of RSV could lead to a decreased economic burden of disease in older adults.

Incorporating studies like the present analysis can enhance health technology assessment (HTA) processes for RSV vaccines, facilitating evidenced-based decision-making. National immunization programs require HTA frameworks that integrate epidemiology, disease burden, and economic analyses [40]. Although estimating the overall value of vaccines and vaccination is complex, the use of evidence-based tools and new economic models is crucial. Current assessments remain limited and require additional health data, such as the findings from this analysis, to improve their accuracy [41].

## 5. Conclusions

Overall, this study reported a substantial economic burden associated with RSV among adults aged ≥ 60 years with at least one RSV hospitalization discharge diagnosis code in Italy. The economic burden was significantly higher in those hospitalized for RSV compared with those hospitalized for any cause and without a diagnosis for RSV. Together, the results emphasize the importance of preventative strategies for RSV, such as vaccination, to reduce the severity of disease symptoms and, in turn, lead to individual and community health benefits, reduced healthcare costs, and greater sustainability of healthcare systems. These data may assist policymakers and healthcare providers in making informed decisions regarding recommendations for and the implementation of RSV vaccination in Italy.

## Figures and Tables

**Table 1:** Mean healthcare resource utilization per patient during the index hospitalization and 12-month follow-up period, stratified by age.

	≥60 Years (*n* = 201)	≥65 Years (*n* = 176)	≥75 Years (*n* = 120)
Hospitalizations (index included)	1.95 (1.6)	1.85 (1.4)	1.73 (1.2)
RSV hospitalizations	1.01 (0.1)	1.01 (0.1)	1.01 (0.1)
ICU admissions	0.05 (0.2)	0.05 (0.2)	0.05 (0.2)
Drug prescriptions	19.38 (17.5)	18.75 (17.3)	17.00 (17.2)
Outpatient services	7.11 (11.7)	6.48 (9.1)	5.08 (7.6)
Specialized visits	2.09 (4.7)	1.77 (3.4)	1.55 (3.5)
Diagnostic/laboratory tests	5.02 (7.8)	4.71 (6.7)	3.53 (5.0)

Data reported as mean (SD) with outliers (defined as values that fall ≥3 SDs from the mean) included. Abbreviations: ICU, intensive care unit; RSV, respiratory syncytial virus; SD, standard deviation.

**Table 2:** Mean direct healthcare costs per patient during the index hospitalization and 12-month follow-up period, stratified by age.

	≥60 Years (*n* = 201)	≥65 Years (*n* = 176)	≥75 Years (*n* = 120)
Hospitalizations (index included)	9156.54 (12,884.39)	8162.50 (10,871.61)	6918.38 (6919.23)
RSV hospitalizations	4892.61 (7089.70)	4314.54 (4829.37)	4135.66 (4504.82)
ICU admissions	793.36 (4491.62)	625.08 (3930.32)	794.49 (4635.72)
Drug prescriptions	1879.36 (4805.24)	1441.62 (2706.53)	1080.92 (2312.87)
Outpatient services	563.45 (1366.15)	468.63 (1077.72)	386.19 (1012.45)
Specialized visits	47.19 (108.39)	40.93 (86.70)	38.19 (93.27)
Diagnostic/laboratory tests	516.26 (1306.18)	427.70 (1049.16)	348.00 (990.92)
**Total cost**	**11,599.35 (15,401.33)**	**10,072.75 (11,680.55)**	**8385.48 (7618.00)**

Data reported as mean (SD) in Euros (EUR) with outliers (defined as values that fall ≥3 SDs from the mean) excluded. Abbreviations: ICU, intensive care unit; RSV, respiratory syncytial virus; SD, standard deviation.

**Table 3:** Mean healthcare resource utilization per patient aged ≥ 60 years during the index hospitalization and 12-month follow-up period, stratified by risk status, comorbidity, and immunocompromised status.

	High-Risk (*n* = 132)	COPD (*n* = 81)	Heart Failure (*n* = 35)	Diabetes (*n* = 48)	Immunocompromised (*n* = 85)
Hospitalizations (index included)	1.99 (1.7)	1.91 (1.6)	2.09 (1.9)	1.90 (1.4)	2.41 (2.1)
RSV hospitalizations	1.02 (0.1)	1.00 (0.0)	1.00 (0.0)	1.02 (0.1)	1.00 (0.0)
ICU admissions	0.06 (0.2)	0.09 (0.3)	0.03 (0.2)	0.06 (0.2)	0.06 (0.2)
Drug prescriptions	20.77 (19.2)	21.28 (19.6)	24.63 (21.5)	21.88 (19.7)	21.21 (19.0)
Outpatient services	6.91 (11.9)	5.26 (6.8)	5.57 (7.8)	5.42 (6.4)	9.75 (15.5)
Specialized visits	2.23 (5.1)	1.58 (2.9)	2.17 (3.5)	1.50 (2.9)	2.95 (6.2)
Diagnostic/laboratory tests	4.67 (7.4)	3.68 (5.1)	3.40 (4.9)	3.92 (4.4)	6.80 (10.2)

Data reported as mean (SD) with outliers (defined as values that fall ≥3 SDs from the mean) included. High-risk patients were defined by the presence of at least one comorbidity of interest during the baseline period. Abbreviations: COPD, chronic pulmonary obstructive disorder; ICU, intensive care unit; RSV, respiratory syncytial virus; SD, standard deviation.

**Table 4:** Mean direct healthcare costs per patient aged ≥ 60 years during the index hospitalization and 12-month follow-up period, stratified by risk status, comorbidity, and immunocompromised status.

	High-Risk (*n* = 132)	COPD (*n* = 81)	Heart Failure (*n* = 35)	Diabetes (*n* = 48)	Immunocompromised (*n* = 85)
Hospitalizations (index included)	9614.87 (12,678.16)	9176.04 (10,761.54)	8097.01 (7889.07)	9044.52 (10,504.44)	12,355.68 (17,677.14)
RSV hospitalizations	5410.77 (8103.04)	5366.04 (7765.23)	3975.13 (2620.25)	5741.98 (8848.05)	5701.09 (8903.05)
ICU admissions	914.54 (4697.52)	1301.67 (5734.03)	18.20 (107.67)	656.56 (3116.50)	821.23 (4308.32)
Drug prescriptions	2186.65 (5609.33)	2076.20 (5688.82)	1538.11 (1519.10)	2148.81 (4703.81)	2956.63 (7130.77)
Outpatient services	467.03 (1055.26)	376.89 (820.33)	284.47 (533.76)	281.88 (449.12)	816.22 (1603.17)
Specialized visits	51.54 (116.97)	33.64 (71.65)	52.43 (105.60)	36.33 (69.94)	61.60 (130.44)
Diagnostic/laboratory tests	415.49 (967.69)	343.26 (782.64)	232.04 (470.66)	245.55 (411.67)	754.63 (1506.31)
**Total cost**	**12,268.55 (15,322.52)**	**11,629.13 (12,883.56)**	**9919.59 (8282.78)**	**11,475.22 (14,153.54)**	**16,128.53 (21,302.39)**

Data reported as mean (SD) in Euros (EUR) with outliers (defined as values that fall ≥3 SDs from the mean) excluded. High-risk patients were defined by the presence of at least one comorbidity of interest during the baseline period. Abbreviations: COPD, chronic pulmonary obstructive disorder; ICU, intensive care unit; RSV, respiratory syncytial virus; SD, standard deviation.

**Table 5:** Mean healthcare resource use and direct healthcare costs among patients hospitalized for RSV and patients hospitalized for any cause (propensity-score-matched analysis).

Mean HCRU (outliers included)			
	≥60 years population hospitalized for any cause (*n* = 1005)	≥60 years RSV-hospitalized patients (*n* = 201)	*p*-value
Hospitalizations (index included *)	1.67 (1.1)	1.95 (1.6)	<0.01
ICU admissions	0.02 (0.2)	0.05 (0.2)	<0.05
Drug prescriptions	18.92 (15.2)	19.38 (17.5)	=0.701
Outpatient services	6.59 (9.2)	7.11 (11.7)	=0.479
Specialized visits	1.71 (3.2)	2.09 (4.7)	=0.154
Diagnostic/laboratory tests	4.87 (7.9)	5.02 (7.8)	=0.809
**Mean direct healthcare costs (outliers excluded)**			
	≥60 years population hospitalized for any cause (*n* = 992)	≥60 years RSV-hospitalized patients (*n* = 195)	*p*-value
Hospitalizations (index included *)	6327.09 (6395.82)	7476.72 (7575.15)	<0.05
ICU admissions	177.83 (1936.50)	817.77 (4558.35)	=0.001
Drug prescriptions	1304.04 (2148.93)	1393.92 (2583.46)	=0.606
Outpatient services	541.92 (1563.60)	498.82 (1190.71)	=0.716
Specialized visits	34.14 (71.84)	45.42 (107.15)	=0.068
Diagnostic/laboratory tests	507.77 (1545.69)	453.40 (1134.00)	=0.641
**Total cost**	**8173.04** **(7198.54)**	**9369.46** **(8437.93)**	**<0.05**

* Index hospitalization was the first RSV hospitalization during the study period for the RSV-hospitalized cohort and the first hospitalization (any cause) during the study period for the control cohort. Data are reported as mean (SD) and direct costs are reported in Euros (EUR). Outliers (defined as values that fall ≥3 SDs from the mean) were included for HCRU results and excluded for direct healthcare cost results. Abbreviations: HCRU, healthcare resource use; ICU, intensive care unit; RSV, respiratory syncytial virus; SD, standard deviation.

## Data Availability

Data used for this publication were generated by CliCon S.r.l. For access to anonymized subject-level data, please contact CliCon S.r.l.

## Supplementary Materials

The following supporting information can be downloaded at: https://www.mdpi.com/article/10.3390/diseases13030068/s1, Figure S1: Study design and patient population for RSV-hospitalized patients (RSV-hospitalized cohort) and patients hospitalized for any cause (control cohort); Table S1: Codes and criteria for the identification of comorbidities and the identification of immunocompromised; Table S2: Baseline demographics for RSV-hospitalized patients, stratified by age; Table S3: Baseline demographics for RSV-hospitalized patients aged ≥ 60 years, stratified by comorbidity and immunocompromised status.
