# Supplementary material for: Healthcare Resource Utilization and Economic Outcomes of RSV-Hospitalized Patients Aged ≥ 60 Years: A Retrospective Cohort Study
Source: Diseases. 2025 Feb 21;13(3):68. doi: 10.3390/diseases13030068 (PMC11941357; doi:10.3390/diseases13030068)
Supplement: Supplementary file 1 [file diseases-13-00068-s001.zip › diseases-3402987-supplementary.pdf]

Supplementary Tables/Figures

Supplementary Table S1. Codes and criteria for the identification of comorbidities and the identification of immunocompromised

**Codes for the identification of comorbidities**	
**Comorbidity**	**Codes and proxies**
COPD	At least two prescriptions of drugs belonging from ATC code R03: drugs for obstructive airway diseases class, in particular: inhaled glucocorticoids (ATC R03BA); adrenergic in combination with corticosteroids or other drugs, excl. anticholinergics (ATC R03AK); selective beta-2-adrenoreceptor agonists (ATC R03AC12, salmeterol, R03AC13, formoterol, R03AC14, clenbuterol, R03AC18, indacaterol, R03AC02, salbutamol, R03AC03, terbutaline, R03AC04, fecosterol, R03AC05, rimiterol); bambuterol (ATC R03CC12); anticholinergics (ATC R03BB); leukotriene receptor antagonists (ATC R03DC)
	At least one hospitalization with primary or secondary discharge diagnosis with ICD-9-CM code 491.X (chronic bronchitis)
	At least one hospitalization with primary or secondary discharge diagnosis with ICD-9-CM code 492.X (emphysema)
	At least one hospitalization with primary or secondary discharge diagnosis with ICD-9-CM code 494.X (bronchiectasis)
	At least one hospitalization with primary or secondary discharge diagnosis with ICD-9-CM code 496.X (chronic airway obstruction, not elsewhere classified)
	An active exemption code 057 (COPD) in "moderate", "severe" and "very severe" clinical stages
Diabetes	Identified by at least two prescriptions of drugs with ATC code A10
	At least one hospitalization with primary or secondary discharge diagnosis with ICD-9-CM code 250 (diabetes mellitus without mention of complication)
	An active exemption code 013 for diabetes
Heart failure	Identified by the presence of at least one hospitalizations with primary or secondary discharge diagnosis with ICD-9-CM code 428.X (congestive heart failure, unspecified)
	An active exemption code 021.428 for heart failure
**Codes and criteria for the identification of immunocompromised**	
**Criteria**	**Codes and proxies**
Hematopoietic stem cell transplant	At least one hospitalization with primary or secondary discharge diagnosis with ICD-9-CM code 41.0 (bone marrow or hematopoietic stem cell transplant)
Solid organ transplant	At least one hospitalization with primary or secondary discharge diagnosis with ICD-9-CM codes: V42.0, 55.6 – kidney, V42.1 – heart, V42.2 – heart valve, V42.3 – skin, V42.5 – cornea, V42.6 – lung, V42.7 – liver, V42.83 –pancreas, V42.84 – intestine
Rheumatoid arthiritus	At least one hospitalization with primary or secondary discharge diagnosis with ICD-9-CM code 714 (rheumatoid arthritis and other inflammatory polyarthropathies)
	An active exemption code 006 for rheumatoid arthritis
Systemic lupus erythematosus	At least one hospitalization with primary or secondary discharge diagnosis with ICD-9-CM code 710.0 (systemic lupus erythematosus)
	An active exemption code 028.710.0 for systemic lupus erythematosus
Inflammatory bowel disease	At least one hospitalization with primary or secondary discharge diagnosis with ICD-9-CM codes 555 (Crohn's disease), 556 (ulcerative colitis)
	An active exemption code 009.555, 009.556 for Crohn disease and ulcerative colitis, respectively

Psoriasis	At least one hospitalization with primary or secondary discharge diagnosis with ICD-9-CM codes 696.1 (other psoriasis)
	An active exemption code 045.696.1 for psoriasis
	At least two prescriptions for antipsoriatic topical drugs (ATC code: D05AA)
Multiple sclerosis	At least one hospitalization with primary or secondary discharge diagnosis with ICD-9-CM code 340 (multiple sclerosis)
	At least two prescriptions of specific medications with ATC codes: L03AB07, interferon beta 1-a, L03AB08, interferon beta 1-b, L03AB13, peginterferon beta 1-a, L03AX13, glatiramer acetate, L04AA31, teriflunomide, N07XX09, dimethyl fumarate, L04AX09, diroximel fumarate, L04AA23, natalizumab, L04AA27, fingolimod, L04AA34, alemtuzumab, L04AA36, ocrelizumab, L04AA40, cladribine, L04AA52, ofatumumab, L04AA50)
	An active exemption code 046.340 for multiple sclerosis
Polymyalgia rheumatica	At least one hospitalization with primary or secondary discharge diagnosis with ICD-9-CM code 725 (polymyalgia rheumatica)
Autoimunne thyroiditis	At least one hospitalization with primary or secondary discharge diagnosis with ICD-9-CM code 245.2 (chronic lymphocytic thyroiditis)
	An active exemption code 056 for autoimmune thyroiditis
Human immunodeficiency virus	At least one hospitalization with primary or secondary discharge diagnosis with ICD-9-CM code 042 (HIV disease); 043 (HIV infection causing other specified conditions); 04 (other HIV infection; 079.53 (HIV type 2; 795.71 (serological evidence not specified of HIV); V08 (infective HIV state)
	At least two prescriptions for drugs with ATC J05AE, protease inhibitors; J05AG, non-nucleoside reverse transcriptase inhibitors; J05AJ, integrase inhibitors; J05AR, antivirals for treatment of HIV infections; J05AX07, enfuvirtide; J05AX09, maraviroc; J05AX23, ibalizumab; J05AX29, fostemsavir
	An active exemption code 020.042 for HIV
End-stage renal disease	At least one hospitalization with primary or secondary discharge diagnosis with ICD-9-CM codes 39.95 (hemodialysis), 54.98 (peritoneal dialysis [primary or secondary procedure]), or specialistic codes 39.95 (insertion of catheter for hemodialysis), 54.98 (peritoneal dialysis)
Cancer (both solid and hematological malignancies)	At least one hospitalization with primary or secondary discharge diagnosis with ICD-9-CM codes: from 140.X to 209.X
	At least two prescriptions of drugs with ATC code L01 (antineoplastic agents)
	An active exemption code 048 for cancer
Immunosuppressive therapy	At least two prescriptions of immunosuppressant (ATC code L04) or systemic corticosteroids [identified by ATC code H02], used for more than 4 consecutive weeks or used at high-dose corticosteroids (i.e., 20 or more mg of prednisone or equivalent per day when administered for 2 or more weeks)
**Codes for invasive hospital procedures**	
**Procedure**	**Code and proxies**
Intubation	Procedural code 96.0X
High-flow oxygen therapy	Procedural code 93.90
Intermittent positive airway pressure	Procedural code 93.91
Other continuous mechanical ventilation	Procedural code 96.7

For the identification of comorbidities or immunocompromised status, adults had to satisfy at least one of the criteria. Abbreviations: ATC, Anatomical Therapeutic Chemical; COPD, chronic obstructive pulmonary disease; HIV, human immunodeficiency virus; ICD-9-CM, International Classification of Diseases, Ninth Revision, Clinical Modification.

Supplementary Table S2. Baseline demographics for RSV-hospitalized patients, stratified by age

	**≥60 years (*n*=201)**	**≥65 years (*n*=176)**	**≥75 years (*n*=120)**
Age, mean (SD)	77.8 (10.4)	80.1 (9.1)	85.0 (6.3)
Male, n (%)	78 (38.8)	61 (34.7)	38 (31.7)
CCI, mean (SD)	1.9 (1.5)	1.9 (1.4)	1.8 (1.4)
Low CCI = 0, n (%)	30 (14.9)	24 (13.6)	18 (15.0)
Medium CCI = 1 or 2, n (%)	117 (58.2)	106 (60.2)	70 (58.3)
High CCI = 3 or 4, n (%)	41 (20.4)	35 (19.9)	26 (21.7)
Very High CCI ≥5, n (%)	13 (6.5)	11 (6.3)	6 (5.0)
**Pre-existing conditions, n (%)**			
COPD	81 (40.3)	71 (40.3)	45 (37.5)
Asthma	8 (4.0)	8 (4.5)	5 (4.2)
Diabetes	48 (23.9)	43 (24.4)	28 (23.3)
Heart failure	35 (17.4)	32 (18.2)	25 (20.8)
Advanced liver disease	4 (2.0)	4 (2.3)	4 (3.3)
Renal disease	30 (14.9)	26 (14.8)	19 (15.8)

Abbreviations: CCI, Charlson Comorbidity Index; COPD, chronic pulmonary obstructive disorder; RSV, respiratory syncytial virus; SD, standard deviation.

Supplementary Table S3. Baseline demographics for RSV-hospitalized patients aged ≥60 years, stratified by comorbidity and immunocompromised status

	**COPD (*n*=81)**	**Heart failure (*n*=35)**	**Diabetes (*n*=48)**	**Immuno-compromised (*n*=85)**
Age, mean (SD)	76.6 (9.9)	80.2 (10.6)	77.1 (10.3)	77.4 (10.0)
Male, n (%)	30 (37.0)	13 (37.1)	20 (41.7)	22 (25.9)
CCI, mean (SD)	2.1 (1.5)	2.7 (1.6)	2.8 (1.6)	2.4 (1.7)
Low CCI = 0, n (%)	5 (6.2)	<4	<4	8 (9.4)
Medium CCI = 1 or 2, n (%)	50 (61.7)	15 (42.9)	22 (45.8)	46 (54.1)
High CCI = 3 or 4, n (%)	19 (23.5)	13 (37.1)	18 (37.5)	22 (25.9)
Very High CCI ≥5, n (%)	7 (8.6)	6 (17.1)	7 (14.6)	9 (10.6)
**Pre-existing conditions, n (%)**				
COPD	81 (100.0)	20 (57.1)	21 (43.8)	39 (45.9)
Asthma	7 (8.6)	<4	<4	5 (5.9)
Diabetes	21 (25.9)	15 (42.9)	48 (100.0)	14 (16.5)
Heart failure	20 (24.7)	35 (100.0)	15 (31.3)	16 (18.8)
Advanced liver disease	<4	<4	<4	3 (3.5)
Renal disease	17 (21.0)	12 (34.3)	10 (20.8)	18 (21.2)

Abbreviations: CCI, Charlson Comorbidity Index; COPD, chronic pulmonary obstructive disorder; RSV, respiratory syncytial virus; SD, standard deviation.

Supplementary Figure S1. Study design and patient population for RSV-hospitalized patients (RSV-hospitalized cohort) and patients hospitalized for any cause (control cohort)

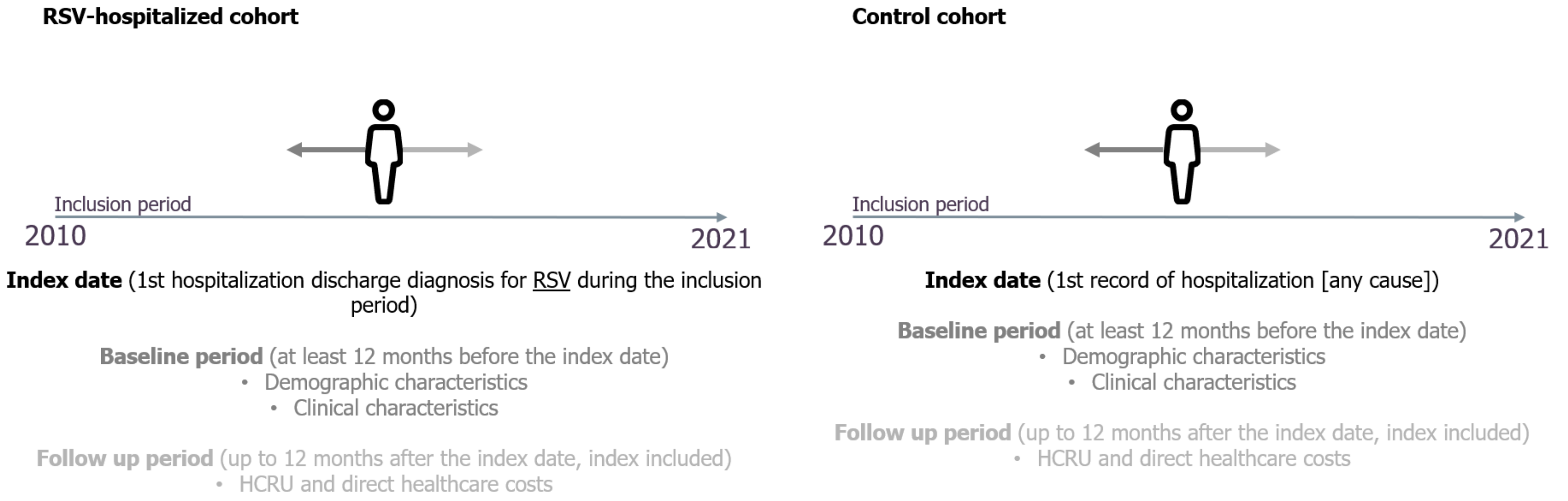

The control cohort (patients hospitalized for any cause) were included in the propensity-score-matched analyses only. Abbreviations: HCRU, healthcare resource utilization; RSV, respiratory syncytial virus.
